# Announcement: 6^th^ International Symposium on Metal Ions in Biology and Medicine

**DOI:** 10.1155/MBD.1999.199

**Published:** 1999


					6th
INTERNATIONAL SYMPOSIUM ON METAL IONS
IN BIOLOGY AND MEDICINE
May 7- 10, 2000
Caribe Hilton Hotel and Convention Center, San Juan, Puerto Rico
The Organizing Committee and the Armed Forces Institute of Pathology invite you to participate
in the 6 International Symposium on Metal Ions in Biology and Medicine, to be held on May
7-10, 2000, in San Juan, Puerto Rico-USA. The objective of the 6th
ISMIBM is to foster exchange
of opinions between professionals and specialists working on analysis, research and applications
of metal ions, trace elements and minerals in biological, biochemical, medical sciences,
toxicology and environmental health. The scientific program, composed of plenary and
concurrent sessions, and poster presentations is designed to promote intensive and productive
dialogue among experts in these fields. A special program with short courses and mini-symposia
have also been organized, featuring specialized areas including toxicology, analysis, pathology,
remediation strategies, and environmental medicine.
Original contributions (oral and/or poster presentations) are invited on the
following themes:
Metals and Environmental Health
Molecular Toxicology of Metals
= Carcinogenicity of Metals
Speciation of Metals and Other Elements
Uses of Metals in Clinical Applications
Metals and Disease: Environmental and Toxicologic Pathology
= Epidemiology and Occupational Health
Metals and Aging
= Metals and Homeostasis
Effects of Low and High Nutritional Trace Element Status
= Metals and Hormone Actions
Metals and Enzyme Activity
= Metals and Chelation Therapy
= Health Effects of Arsenic
= Risk Assessment of Trace Element Status and Health
= Advanced Methods for the Analysis of Trace Elements and Metal Ions
199
Deadlines:
Submission of Abstracts for oral or poster presentations:
Notification of acceptance of Abstracts:
Submission of Full Manuscript:
Early Registration:
Hotel Reservations:
September 15, 1999
October 15, 1999
March 3, 2000
January 14, 2000
April 10, 2000
The 6" ISMIBM will take place in San Juan, Puerto Rico, on the grounds of the Caribe Hilton
Hotel and Convention Center. The language of the Symposium is English.
For additional information and to receive a complete copy of the Second Announcement and
Final Call for Papers with Registration Form, please contact the Symposium Service Office at:
American Registry of Pathology
ATTN" Dr. Jose A. Centeno, Chairman, 6" ISMIBM,
Armed Forces Institute of Pathology
Washington, DC 20306-6000, USA
E-mail: centeno@afip.osd.mil
Telephones: Toll Free (USA only)" 1-800-577-3749; or 1-202-782-2637
Fax: International Toll Free Fax: +1-877-891-3482 or +1-202-782-5020
Websites
On-line Registration to the 6" ISMIBM:
http://www.afip.org/.edu, (click on "Conferences")
General Information"
http://www.afip.o.rg/centeno
200

				

